# Overexpression of complement component C5a accelerates the development of atherosclerosis in ApoE-knockout mice

**DOI:** 10.18632/oncotarget.11180

**Published:** 2016-08-10

**Authors:** Guipeng An, Bo Li, Xiaoman Liu, Mingxiang Zhang, Fei Gao, Yuxia Zhao, Fengshuang An, Yun Zhang, Cheng Zhang

**Affiliations:** ^1^ The Key Laboratory of Cardiovascular Remodeling and Function Research, Chinese Ministry of Education and Chinese Ministry of Health, Shandong University Qilu Hospital, Jinan, Shandong, China; ^2^ Department of Cardiology, Shandong University Qilu Hospital, Jinan, Shandong, China

**Keywords:** complement system, atherosclerosis, C5a, Pathology Section

## Abstract

**Background:**

In this study, we investigated the direct effect of C5a overexpression on atherosclerosis.

**Methods and Results:**

A recombinant adenovirus expressing mouse C5a (Ad-C5a) was constructed and injected intravenously into ApoE^−/−^ mice. After 12 weeks of a high-fat diet, Ad-C5a injection produced more extensive lesions than control adenovirus, and its proathrosclerotic role was significantly blocked by C5a receptor antagonist. Immunohistochemical analysis showed enhanced macrophage infiltration in atherosclerotic regions with C5a overexpression. Trans-well assay revealed C5a receptor-dependent chemotaxis of C5a to macrophages. Furthermore, Ad-C5a overexpression promoted foam cell formation and lipid deposition but reduced collagen content. In addition, with Ad-C5a overexpression, the serum levels of interleukin 6 and tumor necrosis factor α were upregulated.

**Conclusions:**

C5a overexpression could accelerate the development of atherosclerosis in ApoE^−/−^ mice by promoting macrophage recruitment, foam cell formation and inflammatory activation. Furthermore, its proatherogetic role is mediated by the C5a receptor.

## INTRODUCTION

Recent studies have suggested that atherosclerosis is a chronic inflammatory condition of arterial walls, as evidenced by the presence of inflammatory cells, activated immune cells, and cytokines in atherosclerotic lesions [1–3]. As an important part of innate immunity, the components of the complement system, such as iC3d, C5b-9, and complement receptors, were detected in atherosclerotic lesions, and the extensive activation of complement system in vulnerable and ruptured plaques further indicate its involvement in the development of athrosclerosis [4–6].

The anaphylatoxin C5a is a 74-amino acid glycoprotein generated by enzymatically cleaving C5 during the activation of the complement cascade [7]. Acting *via* a classical G protein-coupled receptor (C5a receptor, C5aR) present in immune-inflammatory cells, including monocytes, macrophages, neutrophils, and T cells, C5a mediates immune and inflammatory processes such as increased vascular permeability, spasmogenesis, immune regulation, and release of various inflammatory cytokines and mediators [8, 9]. In addition, C5a is a strong chemoattractant and is involved in the recruitment of many inflammatory cells such as T lymphocytes, eosinophils, neutrophils, and monocytes [10, 11].

Recently, several studies provided clues for the involvement of C5a in atherosclerosis. C5a receptor blockage with C5aR antagonist or anti-C5aR-blocking monoclonal antibody could limit neointimal hyperplasia and inflammatory cell content in a model of wire-induced endothelial denudation [12]. Treatment with a C5a receptor antagonist, PMX53, has been shown to reduce lesion size and lipid content in the plaque by about 40% in apolipoprotein E-knockout (ApoE^−/−^) mice [13]. Immunization of mice with C5aR-derived peptides was effective in reducing early atherosclerotic lesion development [14]. However, the role of C5a in the development of atherosclerosis is still not well understood. In this study, we investigated the direct effect of C5a overexpression on the development of atherosclerosis in ApoE^−/−^ mice.

## RESULTS

### C5a protein is expressed *in vitro* and *in vivo* after adenoviral gene transfer

**Figure 1:**
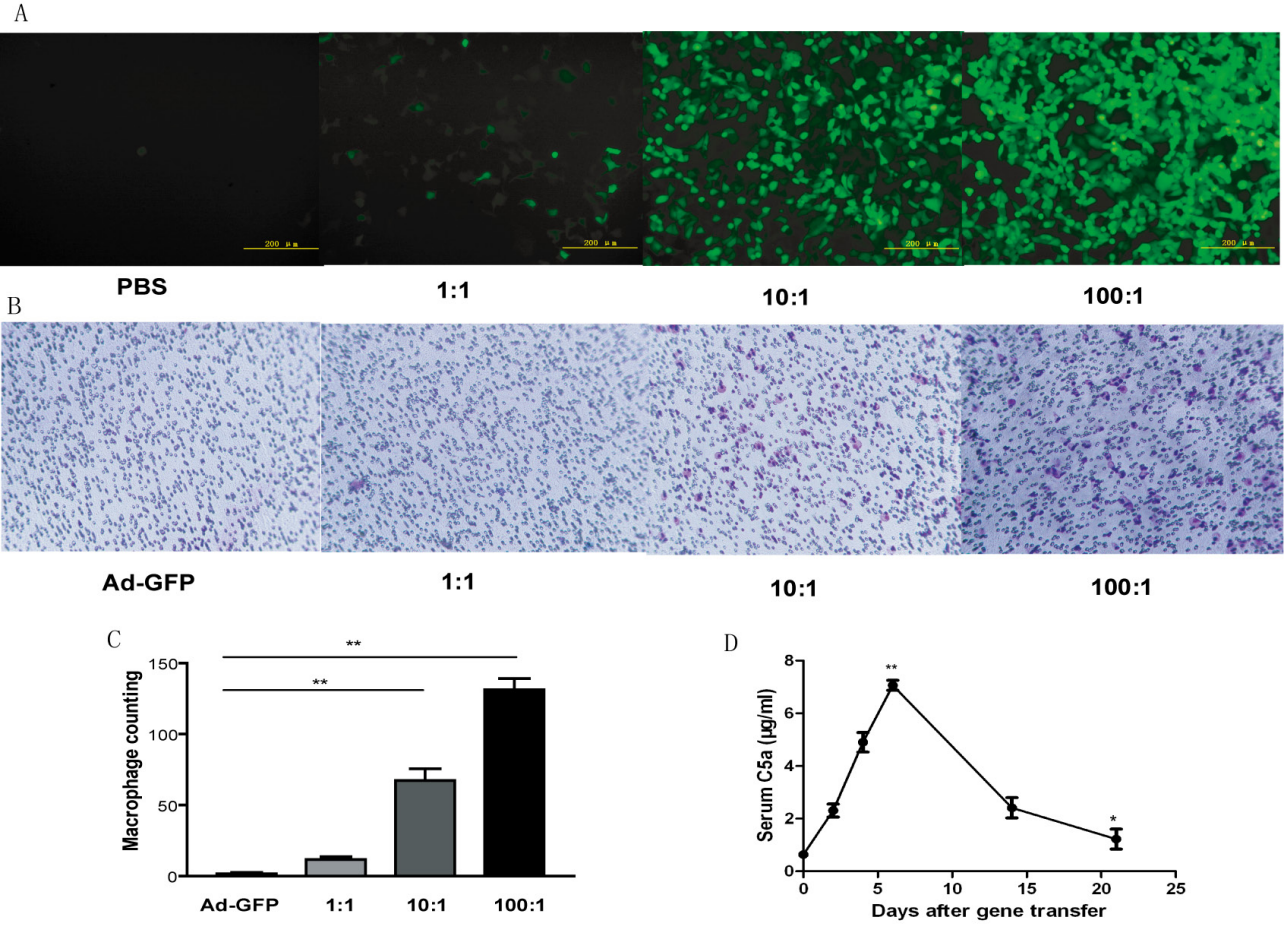
Expression of C5a protein *in vitro* and *in vivo* after adenoviral gene transfer **A.**, Fluorescence images of HEK293 cells after transfection with phosphate buffered saline (PBS) or different multiplicities of infection (MOI) of adenovirus C5a (Ad-C5a) for 24 hr. **B.**, Migration assay of chemotaxis of recombinant C5a to macrophages. HEK293 cells were transfected with Ad-GFP and different MOI (1:1, 10:1 and 100:1) of Ad-C5a. At 24 hr, the supernatants underwent trans-well assay. **C.**, Quantitative analysis of trans-well assay. Data are mean ± SEM from 5 separate fields in each sample from 3 independent experiments. **P < 0.01. **D.**, Detection of recombinant mouse C5a protein in ApoE^−/−^ mouse plasma (*n* = 5). *P < 0.05 and **P < 0.01 *vs* day 0.

To evaluate the efficacy of Ad-C5a gene transfer on protein expression, HEK293 cells were transfected with PBS and different multiplicities of infection (MOI; 1:1, 10:1 and 100:1) of Ad-C5a. Concentration-dependent GFP protein expression was detected after 24 hr (Figure 1A). The function of recombinant C5a was confirmed with trans-well assay. HEK293 cells were transfected with Ad-GFP and different MOI (1:1, 10:1 and 100:1) of Ad-C5a for 24 hr. The supernatant were collected and used in trans-well assay. A concentration-dependent chemotaxis of cell culture supernatant to macrophages was detected (Figure 1B-1C). To test the effect of Ad-C5a gene transfer on serum C5a level, ApoE^−/−^ mice were injected with Ad-C5a. Blood samples were taken at 2, 4, 6, 14, and 21 days after virus injection. Serum C5a level was 8.2-fold higher at 6 days after transfection than at day 0 (*P* < 0.05). At 21 days, C5a concentration was 1.7 fold higher than at day 0 (*P* < 0.01, Figure 1D).

### C5a overexpression accelerated the development of atherosclerosis

**Figure 2:**
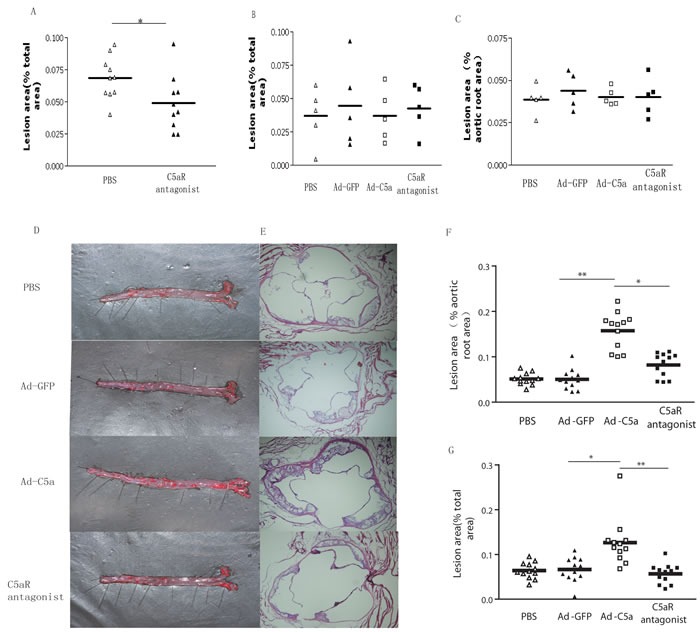
Efffect of C5a overexpression on atherosclerotic lesions in ApoE^−/−^ mice after a 12-week high-fat diet **A.**, Quantitative analysis of atherosclerotic lesions by *en face* staining after C5a receptor antagonist treatment. **B.** and **C.**, Quantitative analysis of atherosclerotic lesions by *en face* staining (B) and in aortic roots (C) after a high fat diet for 8 weeks. **D.** and **E.**, Representative photomicrographs showing atherosclerotic lesions on the surface of aorta (*en face* staining, D) and aortic roots (E). **F.** and **G.** Quantitative analysis of atherosclerotic lesions by *en face* staining (G) and in aortic roots (F). Data are mean±SEM in 6 sections for each mouse sample. N = 12 per group. *P < 0.05, **P < 0.01.

To evaluate the role of C5a under a pathological conditon, 8-week-old male mice were given PBS or C5a receptor antagonist. As shown in Figure 2A, C5a receptor antagonist inhibited the development of atherosclerosis in ApoE^−/−^ mice. To determine the effect of C5a gene transfer on the ability of the high-fat diet to induce atherosclerosis in ApoE^−/−^ mice, we infused a subset of mice fed a high fat diet for 8 weeks with PBS, Ad-GFP, Ad-C5a, or Ad-C5a plus AcF [OPdChaWR]. Mice were sacrificed and the size of atherosclerotic lesions was analyzed at the beginning of treatment or four weeks later. No difference was found between either group before the treatment (Figure 2B, 2C). Four weeks later, lesion size in Ad-C5a group was greater than Ad-GFP group by *en face* staining (10.02 ± 1.12% *vs*. 6.64± 0.8%, *P* = 0.02; Figure 2D, 2G) or aortic root section analysis (12.23± 1.89% *vs*. 5.88± 0.89%, *P* < 0.01; Figure 2E, 2F), but no difference was found between PBS and Ad-GFP group (*P* > 0.05; Figure 2). To investigate whether the C5a receptor was involved in this process, a group of ApoE^−/−^ mice were treated with both Ad-C5a and C5aR antagonist, AcF [OPdChaWR]. Atherosclerotic lesion in C5aR antagonist group was reduced as compared with the Ad-C5a group, by both *en face* staining (5.68± 0.64% *vs*. 10.02 ± 1.12%, *P* < 0.01; Figure 2D, 2G) and aortic root section analysis (6.93 ± 0.87% *vs*. 12.23± 1.89%, *P* < 0.05; Figure 2E, 2F), and no difference in lesion size was found between the C5aR antagonist group and Ad-GFP gourp (*P*> 0.05; Figure 2), therefore proatherosclerotic role of C5a was mostly mediated by the C5a receptor.

### C5a overexpression leads to macrophage accumulation in atherosclerotic lesions

**Figure 3:**
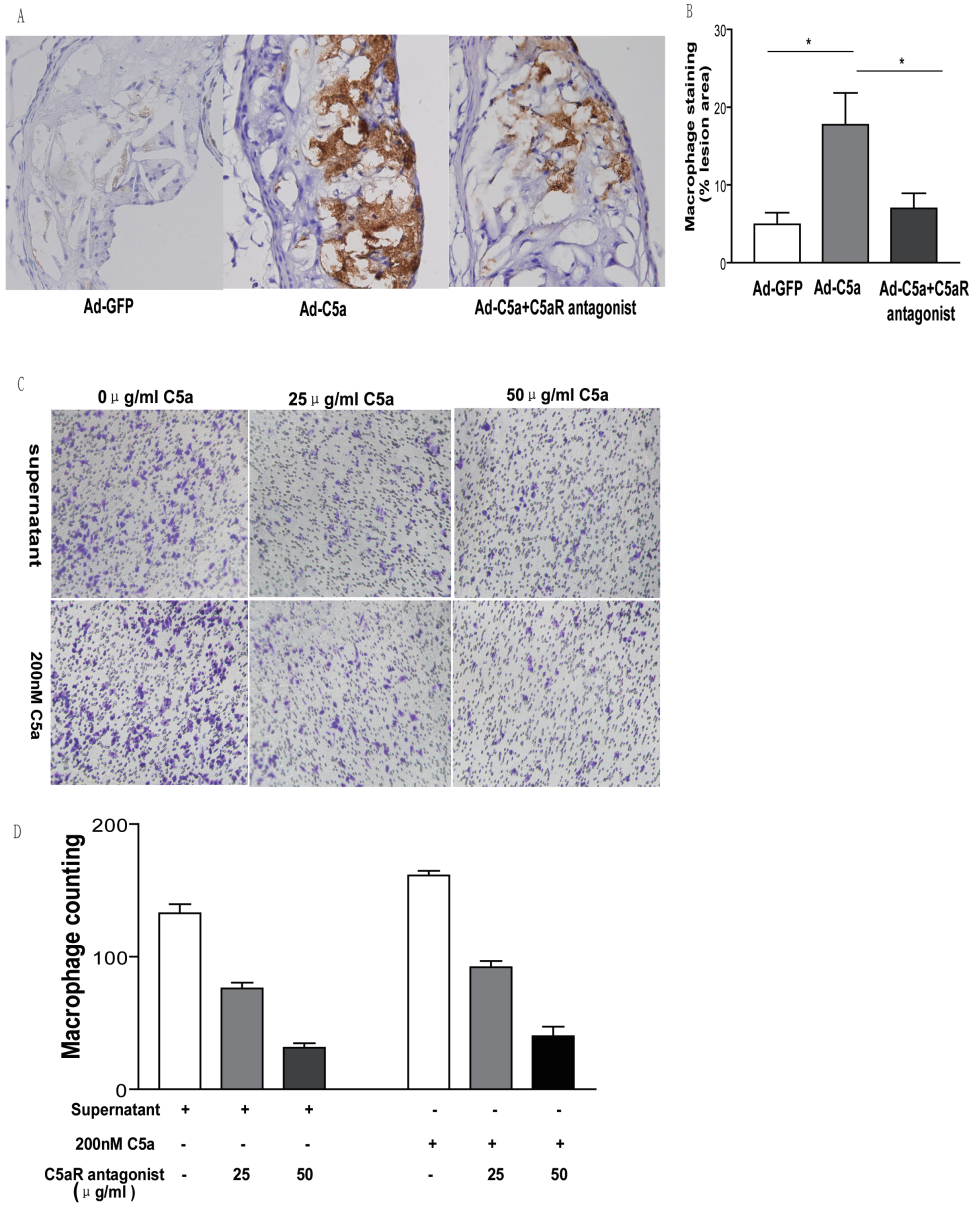
Effect of C5a overexpression on macrophage recruitment **A.**, Representative photomicrographs of atherosclerotic plaque stained for macrophages in the aortic root of ApoE^−/−^ mice. **B.**, Morphometric analysis of stained areas for macrophages. Data are mean±SEM percentage positive area to total lesion area in 6 sections for each mouse. *N* = 8 per group. *P < 0.05. **C.**, Migration assay of chemotaxis of 200 nM C5a or cell culture supernatant to macrophages. HEK293 cells were treated with 100:1 MOI Ad-C5a for 24 hr. The supernatant, 200 nM C5a and different concentrations of C5a receptor antagonist were used in the trans-well assay. **D.**, Quantitative analysis of trans-well assay. Data are mean ± SEM from 5 separate fields in each sample from 3 independent experiments. **P < 0.01.

C5a is known as a strong chemotactic agent for macrophages [15]. To test whether the proatherosclerotic role of C5a was mediated through chemotaxis, aortic root sections were stained for macrophages. Immunohistochemistry showed that macrophage accumulation in Ad-C5a group was higher than that in Ad-GFP group or C5aR antagonist group (*P* < 0.05, Figure 3A, 3B), but no difference in macrophage content was found between Ad-GFP group or C5aR antagonist group (*P* > 0.05, Figure 3A, 3B), which means that C5a overexpresion accelerated macrophage accumulation in atherosclerotic lesions and this role could be blocked by C5aR antagonist. To confirm these findings, we performed *in vitro* migration assay and found prominent macrophage chemotaxis of recombinant mouse C5a and cell culture supernatant of HEK293 cells treated with 100:1 MOI Ad-C5a for 24 hr (Figure 3C, 3D). C5aR antagonist was used to further investigate whether C5aR was involved during macrophage chemotaxis. The macrophage counting in filter was significantly lower in cells treated with both supernatant and 25μg/ml C5aR antagonist than those with only supernatant (*P* < 0.01, Figure 3C, 3D). A significant lower macrophage counting was found in cells treated with both supernatant and 50μg/ml C5aR antagonist than either of other two groups (*P* < 0.01, Figure 3C, 3D). Similar results were found in the experiments carried out with mouse C5a and C5aR antagonist. Therefore, C5a overexpression accelerated macrophage recruitment to atherosclerotic lesions through C5a-receptor-mediated chemotaxis.

### Effect of C5a overexpression on lipid deposition in atherosclerotic lesions

**Figure 4:**
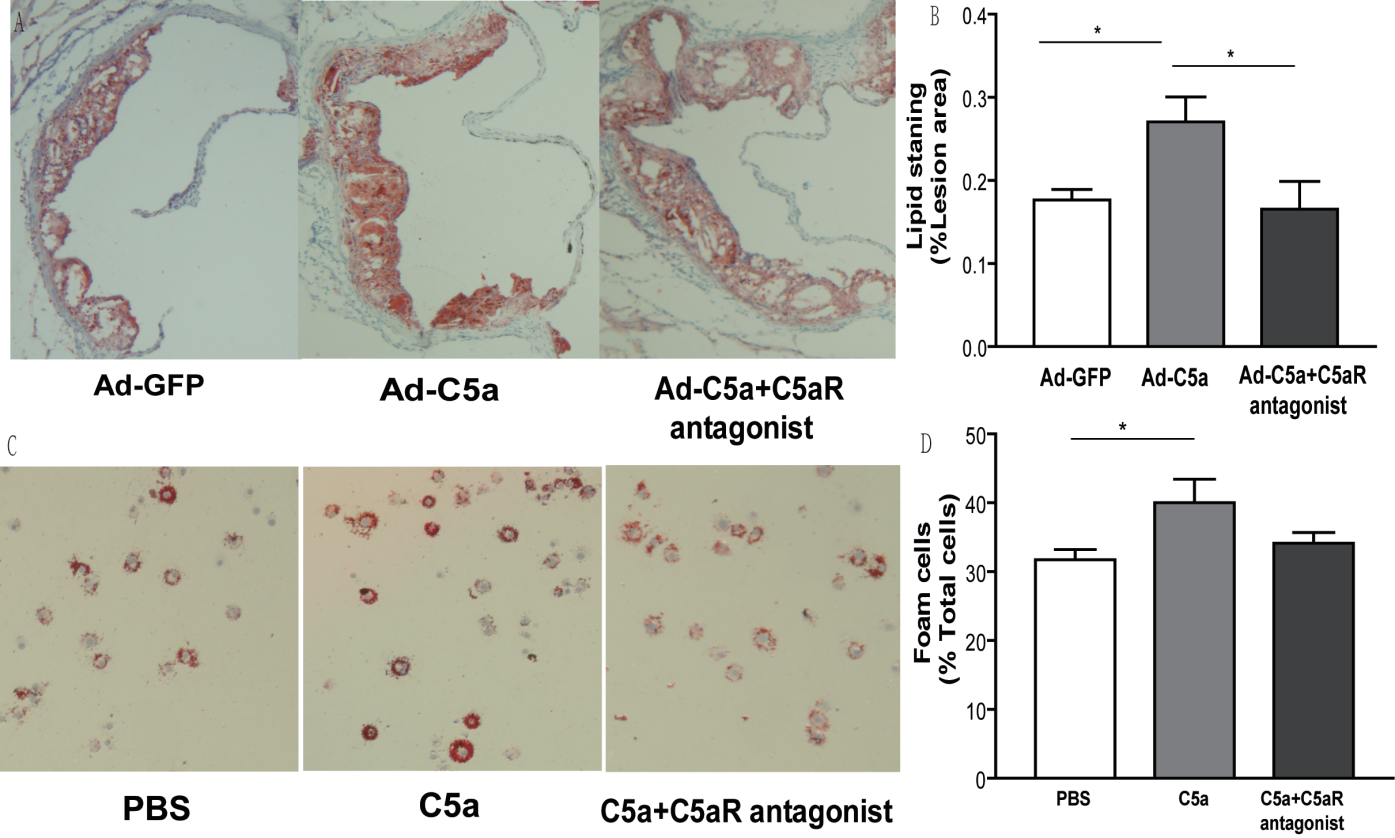
Effect of C5a overexpression on lipid deposition **A.**, Representative photomicrographs of atherosclerotic plaques from the aortic root of ApoE−/− mice stained with Oil-red O for lipids. **B.**, Morphometric analysis of stained areas. Data are mean±SEM percentage positive area to total lesion area in 6 sections for each mouse. *N* = 8 per group. *P < 0.05. **C.**, Representative images of foam cell formation on co-stimulation with oxidized LDL, 50 mg/mL, for 24 hr, and **D.**, quantitative analysis for foam cell formation. 10 microscopic fields were counted from 4 different slides for the same treatment. Data are mean±SEM. *P < 0.05.

As shown in Figure 4, A higher lipid content was observed in atherosclerotic lesion from mice treated with C5a as compared to the control group illustrated with more oil red O staning (*P* < 0.05; Figure 4A, 4B). With the treatment of C5aR antagonist, the content of lipid decreased significantly, compared with those in Ad-C5a alone group (*P* < 0.05; Figure 4A, 4B), and no difference in lipid content was found between Ad-GFP group and C5aR antagonist group. In addition, the influence of C5a on foam cell formation was also tested. After co-stimulation with 50 mg/mL oxLDL for 24 hr, C5a treatment lead to more foam cell formation than with PBS, but this effect was partly blocked by C5a receptor antagonist treatment (Figure 4C, 4D).

### Effect of C5a overexpression on smooth muscle cell proliferation and collagen deposition

**Figure 5:**
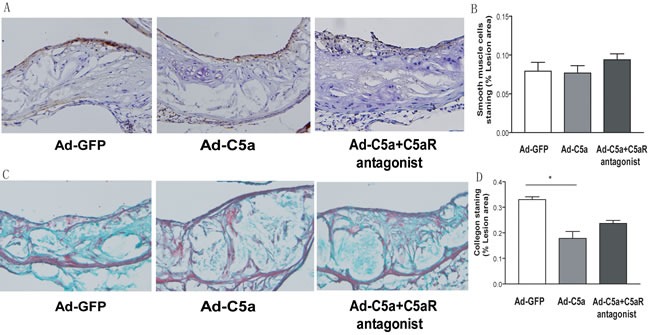
Collagen deposition and smooth muscle cell proliferation in mice **A.**, Representative photomicrographs of atherosclerotic plaques stained for smooth muscle cells in the aortic root of ApoE−/− mice. **B.**, Morphometric analysis of the stained areas for smooth muscle cells. **C.** Representative photomicrographs of atherosclerotic plaques stained for collagen in the aortic root of ApoE−/− mice.**D.**, Morphometric analysis of the stained areas for collagen. Data are mean±SEM percentage positive area to total lesion area in 6 sections for each mouse. *N* = 8 per group. *P < 0.05.

We also carried out immunohistochemistrycal analysis to determine the degrees of collagen deposition and smooth muscle cell proliferation. As shown in Figure 5A and 5B, the content of smooth muscle cell was not statistically different among the 3 groups of mice(*P* > 0.05; Figure 5A, 5B), but a decreasing trend could be found in the Ad-C5a group. However, content of collagen in Ad-C5a group was significant lower than Ad-GFP group *P* < 0.05, Figure 5C, 5D), which suggests that C5a overexpression reduced the deposition of collagen.

### Body weight, serum cholesterol and inflammatory cytokines in mice

**Figure 6:**
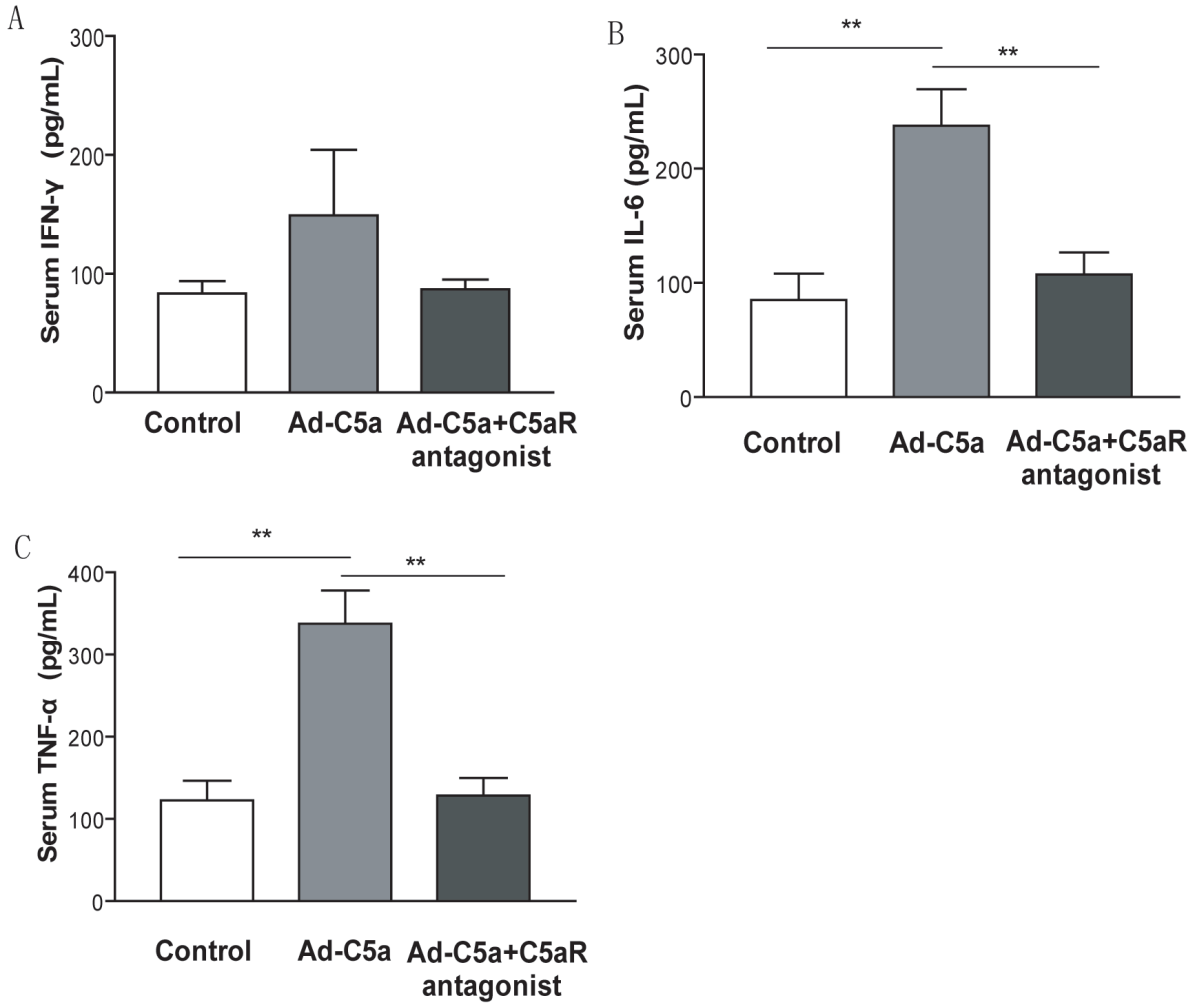
Effect of C5a overexpression on inflammatory cytokines expression Serum from blood samples was taken 14 days after Ad-C5a injection. ELISA of the serum level of interferon γ (IFN-γ) **A.**, interleukin 6 (IL-6) **B.** and tumor necrosis factor α (TNF-α) **C.** *N* = 5 mice per treatment group. Data are means ± SEM. **P < 0.01.

Body weight, total cholesterol, LDL-c, HDL-c, and serum glucose did not differ among the 4 groups of mice (*P* > 0.05; Table 1). To determine whether exacerbated atherosclerosis with Ad-C5a treatment was correlated with increased inflammatory cytokines expression, we analyzed the plasma level of IL-6, IFN-γ and TNF-α in mice. At 14 day after Ad-C5a injection, we chose 5 mice randomly from each treatment group to obtain serum for ELISA assay. Serum IL-6 and TNF-α level in serum were significantly higher in Ad-C5a group, than either Ad-GFP group or C5aR antagonist group (*P* < 0.01; Figure 6), but no difference was found between Ad-GFP group and C5aR antagonist group (*P* > 0.05; Figure 6). However, no difference in serum IFN-γ was detected among the 3 groups of mice (*P* > 0.05; Figure 6).

**Table 1:** Body weight and serum cholesterol level of ApoE^−/−^ mice

	PBS	Ad-GFP	Ad-C5a	C5aR antagonist
Body weight (g)	25.86±0.68	26.03±0.52	25.05±0.51	25.45±0.59
Total cholesterol (mM)	9.27±0.74	9.44±0.87	8.46±0.71	9.66±0.64
HDL cholesterol (mM)	3.02±0.19	3.04±0.11	3.02±0.11	3.09±0.10
LDL cholesterol (mM)	6.35±0.37	6.32±0.50	5.88±0.49	6.38±0.41
Triglyceride (mM)	0.32±0.05	0.33±0.04	0.32±0.02	0.31±0.04
Glucose (mM)	2.88±0.16	2.85±0.18	2.76±0.15	2.99±0.17

PBS, phosphate buffered saline; Ad adenovirus. Data are mean±SEM

## DISCUSSION

Recently, both C5a and its receptors were found abundantly expressed in atherosclerotic lesions [14, 16], and C5a receptor blockage led to significantly reduced lesion size and lipid content in the plaque [13], but little is known about the role of this proinflammatory mediator in atherogenesis. We aimed to determine whether an increase in C5a protein could accelerate the progression of atherosclerosis *in vivo*. We used an adenoviral vector to overexpress C5a in ApoE^−/−^ mice to determine the effects of a brief elevation in C5a protein in established atherosclerosis. Elevated levels of C5a promoted the development of atherosclerotic lesions, which provides direct evidence that C5a contributes to the process of atherosclerosis.

Firstly, we found that Ad-C5a overexpression led to significantly increased lesions in both the aortic surface and aortic roots of ApoE^−/−^ mice fed a high fat diet. A number of studies have implicated the involvement of the complement system in the pathogenesis of atherosclerosis [17]. C3 deficiency was associated with increased lipid-positive lesions in the mouse aorta and altered plasma lipid profile [18, 19]. Rabbits deficient in C6 showed smaller atherosclerotic lesions than controls with a fully functional complement system after a cholesterol-rich diet [20]; C5 or factor B deficiency had no effect on atherosclerosis in ApoE^−/−^ mice [19, 21]. More recently, C1q deficiency caused larger atherosclerotic lesions in LDL-receptor-knockout mice, which suggests a role of C1q in the disposal of dying cells [22]. Taken together, these results suggest a variable and complex role of complement components in atherosclerosis, so studying efforts to better understand its role in atherosclerosis is important. Our data showed that higher levels of C5a promoted the development of atherosclerotic lesions, which provides the fist direct evidence that C5a contributes to the process of atherosclerosis and facilitates the understanding of the role of complement system in this pathophysiological process.

Secondly, we provided evidence to illustrate that the proatherosclerotic role of C5a was mediated by the C5aR. After 12 weeks of a high fat diet, treatment with C5aR antagonist significantly reduced the lesion area, as compared with Ad-C5a alone. The C5a receptor, also known as C5a receptor 1 or CD88, belongs to the rhodopsin-like receptor superfamily, characterized by 7-hydrophobic transmembrane helical regions connected by 3 extracellular and 3 intracellular loops [14, 23]. The receptor is expressed by most of the cell types within human and mouse atherosclerotic plaque, with predominant expression in macrophages but not in normal arteries [13, 28]. The binding interaction of C5a to its receptor CD88 leads to a variety of consequences, including the chemoattraction of inflammatory cells and induction of the “cytokine storm,” including the production of cytokines. Our data showed that the proatherogetic role of C5a could be blocked with C5aR antagnosit, indicating specific C5a-C5aR interaction is important in the development of atherosclerosis.

In addition, our work also provided another evidence for the proatherogetic mechanism of C5a. C5a is considered as the strongest chemoattractant for macrophages, and its receptors are expressed throughout the plaque, with most colocalized with macrophages [13, 24]. As a result, its proatherogetic role may be mediated by recruitment of macrophages to the plaque. Our immunostaining analysis revealed an increased macrophage content in atherosclerotic lesions with Ad-C5a treatment, so C5a maybe act as a chemoattractant in atherogenesis. Previous *in vitro* study showed that C5a-induced chemotaxis could be inhibited by the C5a receptor antagonist [25], and our *in vivo* data confirmed this conclusion, with macrophage content decreased with C5a antagonist treatment as compared with Ad-C5a alone. The data from trans-well assay showed that C5a has a prominent chemotaxis for macrophage and C5aR antagonist has a dose-dependent inhibitory function on it. Those data revealed that the C5a may accelerate the development of atherosclerosis through C5aR-mediated macrophage chemotaxis.

Besides, we also found that treating ApoE^−/−^ mice with Ad-C5a significantly increased lipid content within the atherosclerotic lesions and this role could be blocked by C5aR antagonist treatment, so C5a may act by increasing C5aR-mediated lipid uptake in the plaque. C5a is known to upregulate the production of TNF-α [26, 27], a cytokine known to induce scavenger receptor expression and oxLDL uptake [28]. To confirm this observation, we tested the effect of C5a on foam cell formation and found that C5a promoted macrophage differentiation into foam cells *in vitro*. In addition, we tested the effect of Ad-C5a injection on inflammatory cytokine expression and found a C5aR-related upregulated plasma level of IL-6 and TNF-α, so the proatherogetic role of C5a may be mediated in part by C5aR-related inflammatory cytokine production.

In conclusion, our study demonstrates that C5a overexpression accelerates the development of atherosclerotic lesions in ApoE^−/−^ mice by promoting macrophage recruitment, foam cell formation and inflammatory activation. Furthermore, its proatherogetic role is mediated by the C5a receptor.

## MATERIALS AND METHODS

### Chemicals and reagents

HEK293 cells and RAW264.7 macrophages were obtained from ATCC (Rockville, MD). Recombinant mouse C5a and Oil-red O were from Sigma (St Louis, MO). The 24-well chemotaxis chamber with 8-μm pore size was from BD Biosciences (Oxford, UK). The C5a receptor antagonist AcF [OPdChaWR] was synthesized by GL Biochem (Shanghai). We used mouse interleukin 6 (IL-6), interferon γ (IFN-γ) and tumor necrosis factor α (TNF-α) ELISA kits from eBioscience (San Diego, CA) and a C5a ELISA kit from Cloud-Clone (Houston, TX). Rat anti-mouse monoclonal monocyte/macrophage antibody was from AbD Serotec (Oxford, UK). Rabbit anti-mouse α-smooth muscle actin (α-SMA) antibody was obtained from Abcam (Cambridge, UK). 3,3′-diaminobenzidine staining kit was purchased from ZSGB-Bio (Beijing).

### Generation of recombinant adenovirus

The IFN-γ gene signal sequence (IFN-SS) has been shown to can efficiently induce the production of complement C5a protein [29, 30]. Accordingly, we synthesized the following mouse IFN-SS-C5a DNA with a NdeI site at the 5′ end and HindIII site at the 3′ end: 5′ - ATGAACGCTACACACTGCATCTTGGCTTTGCA GCTCTTCCTCATGGCTGTTTCTGGCTG TTACTGCAACCTGCATCTCCTAAGGC AGAAAATAGAAGAACAAGCTGCT AAGTACAAACATAGTGTGCCAAAG AAATGCTATGACGGAGCCCGAGTGAA CTTCTACGAAACCTGTGAGGAGCG AGTGGCCCGGGTTACCATA GGCCCTCTCTGCATCAGGGCCTTCAACGA GTGCTGTACTATTGCGAACAAGATCCG AAAAGAAAGCCCCCATAAACCTGTCCAACTGGG AAGGTAG - 3′. The IFN-SS-C5a sequence was subcloned into the adenovirus shuttle plasmid vector pDC316-CMV-EGFP, which contains a cytomegalovirus promoter and DNA sequence for Green Fluorescent Protein (GFP). Recombinant adenovirus C5a (Ad-C5a) was generated with pBHGlox_E1,3Cre and the shuttle plasmid by homologous recombination and amplified in HEK293 cells. A recombinant adenovirus encoding enhanced GFP (Ad-GFP) was used as a control. Large-scale viral vectors were purified by CsCl ultracentrifugation and stored at −80°C. The construction of Ad-C5a and Ad-GFP was sourced commercially (Invitrogen, Shanghai).

### Cell transfection analysis

HEK293 cells at low passage number were cultured in Dulbecco's modified Eagle's medium (DMEM) supplemented with 10% (v/v) fetal bovine serum (FBS), 1% glutamine and 1% penicillin/streptomycin. Cells at 90% confluence were transfected with phosphate buffered saline (PBS), Ad-GFP, or Ad-C5a for 24 hr, then viewed under a fluorescence microscope or supernatant was collected for trans-well assay.

### Trans-well assay

Raw264.7 macrophages were cultured in DMEM supplemented with 10% FBS, 1% glutamine and 1% penicillin/streptomycin. To assess macrophage migration, cells (1 × 10^5^/well) were cultured and seeded in the upper layer of a chemotaxis chamber, and the lower chamber contained cell culture supernatant, mouse C5a, or C5a receptor antagonist diluted with DMEM. After incubation for 8 h at 37°C in a 5% CO_2_ incubator, non-migrating cells were removed from the upper surface of the filter by use of a cotton swab and the filter was fixed and stained with 0.1% crystal violet. Photographs were taken and cells were counted in 5 random fields.

### Animals and treatment

Homozygous male ApoE^−/−^ mice (on a C57BL/6J background) were obtained from Peking University (Beijing) and maintained with standard lighting (12:12-h light-dark cycle) and temperature (20°C-22°C) with food and water freely available. The housing and care of the animals and all procedures were performed with the approval of the Animal Care and Use Committee, Shandong University.

As for the test of C5a protein expression, 8-week-old male ApoE^−/−^ mice were injected intravenously with Ad-C5a (2×10^9^ plaque-forming units [pfu] in 200 μL PBS). Blood samples were taken 2, 4, 6, 14, and 21 days later from the retro-orbital plexus of each mouse.

To evaluate the role of C5a under a pathological conditon, 8-week-old male mice were given PBS or C5a receptor antagonist, AcF [OPdChaWR], administered in drinking water (about 1 mg/kg/d) plus injections every weeks (3 mg/kg subcutaneously). Those mice were fed a high-fat diet (0.25% cholesterol and 15% cocoa butter) for 12 weeks and the aortas were analyzed.

To determine the effect of C5a on atherosclerosis, 8-week-old male mice were fed a high-fat diet. Eight weeks later, mice were randomly divided into 4 groups for intravenous injection treatment: PBS group, Ad-GFP group, Ad-C5a group and C5a receptor antagonist group. They were injected intravenously with PBS (200 μL, PBS group), Ad-GFP (2×10^9^ pfu in 200 μL PBS, Ad-GFP group) and Ad-C5a (2×10^9^ pfu in 200 μL PBS, Ad-C5a group). Mice in C5a receptor antagonist group received both Ad-C5a (2×10^9^ pfu in 200 μL PBS) and C5a receptor antagonist, AcF [OPdChaWR], administered in drinking water (about 1 mg/kg/d) plus injections every weeks (3 mg/kg subcutaneously). At the end of the 12^th^ week, the mice from each group were fasted overnight and then sacrificed. Blood was collected by vena cava nicking, and the aorta was perfused with cold PBS by inserting a cannula into the left ventricle. Perfused aortas were dissected from the aortic arch to the iliac bifurcation.

### Processing and analysis of the aorta

The aortas and aortic root sections were prepared and analyzed as described [31]. Aortas were pinned onto plates and stained with Oil-red O. The extent of *en face* atherosclerosis was expressed as the proportion of the aorta surface covered by positive staining. Aortic root sections were prepared and stained with Oil-red O. The extent of aortic root atherosclerosis was expressed as the proportion of aortic root covered by lesions. Images were captured digitally with use of a video camera and analyzed by use of Image Pro Plus (Media Cybernetics). Acquisition of images and analysis of lesions were performed in a blinded fashion.

### Histological analysis of aortic lesions

Tissue samples were stained with hematoxylin and eosin and Oil-red O with standard protocols. Masson trichrome staining was performed as described [32]. For immunohistochemistry, sections were fixed in acetone, rehydrated in PBS, treated with 0.3% H_2_O_2_ in PBS to quench endogenous peroxides, blocked with 5% goat serum, then stained with specific antibodies. Tissue sections were incubated with primary antibodies overnight at 4°C and appropriate biotinylated secondary antibodies for 1 hr at 37°C. A 3,3′-diaminobenzidine staining kit was used to visualize the primary antibody, then sections were rinsed in water and counter-stained with hematoxylin. Data were analyzed by use of ImagePro-Plus.

### Serum sample analysis

Total plasma levels of cholesterol, triglycerides, low-density lipoprotein cholesterol (LDL-c), and high-density lipoprotein cholesterol (HDL-c) were measured using commercially available agents (Zhejiang Dongou Biotechnology, Wenzhou, China). Elisa was conducted according to manufacturer's instruction.

### Macrophage foam cell analysis

Raw264.7 cells were treated with PBS, C5a (200nM) or C5a (200nM) plus Ca5 receptor antanogist (50μg/ml) along with oixidized LDL (50μg/mL) (oxLDL; Guangzhou, China) for 24 hr. After being fixed with 4% paraformaldehyde, cells were stained with 0.5% Oil-red O and counter-stained with hematoxylin. Foam cells were photographed under a microscope, and at least 10 microscopic fields were counted from 4 different slides for the same treatment for quantification.

### Statistical analysis

Data are shown as mean ± SEM. Variables with skewed distribution were analyzed by Mann-Whitney test. For normally distributed data, differences between 2 groups were assessed by unpaired Student's *t* test, and comparison of multiple groups involved use of ANOVA. *P* < 0.05 was considered statistically significant.
